# The Traditional Mongolian Medicine Qiqirigan-8 Effects on Lipid Metabolism and Inflammation in Obesity: Pharmacodynamic Evaluation and Relevant Metabolites

**DOI:** 10.3389/fphar.2022.863532

**Published:** 2022-06-15

**Authors:** Narenmandula, Hongmei, Xiaoqing Ding, Kexin Li, Hashentuya, Dezhi Yang, Wendurige, Rui Yang, Dandan Yang, Tana, Haisheng Wang, Eerdunduleng, Tegexibaiyin, Changshan Wang, Xilinqiqige Bao, Menggenduxi

**Affiliations:** ^1^ School of Traditional Mongolian Medicine, Inner Mongolia Medical University, Hohhot, China; ^2^ School of Basic Medical Sciences, Inner Mongolia Medical University, Hohhot, China; ^3^ School of Life Sciences, Inner Mongolia University, Hohhot, China; ^4^ Innovative Mongolian Medical Engineering Research Center, Inner Mongolia International Mongolian Hospital, Hohhot, China; ^5^ Affiliated Hospital, Inner Mongolia Minzu University, Tongliao, China; ^6^ Medical Innovation Center for Nationalities, Inner Mongolia Medical University, Hohhot, China

**Keywords:** traditional Mongolian medicine Qiqirigan-8, pharmacodynamic evaluation, metabolites, obesity, anti-inflammation, optimize lipid metabolism

## Abstract

**Objective:** Traditional Mongolian Medicine Qiqirigan-8 (MMQ-8) is a Chinese botanical drug with effective pharmacological properties in obesity. However, the pharmacological mechanism of MMQ-8 remains unclear. This study aimed to determine the active metabolites of MMQ-8 and its therapeutic effects on lipid metabolism and inflammation.

**Methods:** The active metabolites of MMQ-8 were identified by ultrahigh-performance liquid chromatograph Q extractive mass spectrometry (UHPLC-QE-MS) assay and network analysis. An obesity rat model induced by high-fat diet was used in the study. Serum levels of lipids and inflammatory factors were detected using biochemical analysis and enzyme-linked immunosorbent assay (ELISA). Pathological analysis of liver tissues and arteries was conducted with hematoxylin and eosin (H&E) staining and immunohistochemistry. Protein expression of the tumor necrosis factor (TNF) signaling pathway was investigated by Western-blot. Simultaneously, bone marrow cells were used for RNA sequencing and relevant results were validated by cell culture and quantitative real-time polymerase chain reaction (RT-qPCR).

**Results:** We identified 69 active metabolites and 551 target genes of MMQ-8. Of these, there are 65 active metabolites and 225 target genes closely related to obesity and inflammation. *In vivo*, we observed that MMQ-8 had general decreasing effects on body weight, white adipose tissue weight, and serum lipids. MMQ-8 treatment notably decreased the liver function markers and hepatic steatosis, and significantly decreased inflammation. In serum, it notably decreased TNF-α, interleukin (IL)-6, and inducible nitric oxide synthase (INOS), while elevating IL-10 levels. MMQ-8 treatment also significantly inhibited proteins phosphorylation of nuclear factor-kappa B inhibitor alpha (IκBα), mitogen-activated protein kinase (p38), extracellular regulated kinase 1/2(ERK1/2), and stress-activated protein kinase/c-Jun N-terminal kinase (SAPK/JNK), and decreased vascular endothelium damage and macrophage infiltration and polarization to M1. These findings coincide with the RNA-sequencing data of bone marrow cells and results of *in vitro* experiments.

**Conclusion:** We determined the pharmacological actions and relevant metabolites of MMQ-8 in obesity for the first time. Our study revealed MMQ-8 can optimize lipid metabolism and reduce chronic inflammation in obesity. However, more in-depth research is needed, for example, to understand the principle of compound compatibility and the inhibition effects on hepatic steatosis, T cell differentiation, and inflammatory signal transduction.

## Introduction

Obesity is a chronic metabolic disease that has become a major health problem worldwide. Furthermore, it is a significant risk factor for insulin resistance, Type 2 diabetes (T2D), non-alcoholic fatty liver disease (NAFLD), cancer, and cardiovascular diseases (CVD) (Malik et al., 2020). In the clinic, statins are commonly used to lower blood lipids and control the occurrence of secondary disease (Tramacere et al., 2019; Cai et al., 2021). However, there are still no radical treatments for obesity and related diseases. Therefore, it is necessary to continue exploring ways to reduce and address the secondary diseases associated with obesity.

Numerous studies indicate that obesity is not only weight gain and abnormal lipid metabolism but also includes systemic chronic low-grade inflammation that has a critical role in the initiation and development of several human illnesses (Furman et al., 2019). In obesity, adipose tissue is an important initiator of the inflammatory response (Heijden et al., 2015). Studies have shown that hypertrophy, hyperplasia, and oxygen depletion of adipose tissue will result in adipocyte dysfunction and inflammation, and the secretion of cytokines such as hypoxia-inducible factor 1 (HIF-1), interleukin (IL)-6, IL-1β, tumor necrosis factor (TNF)-α, and C-C motif chemokine 2 (CCL2). These cytokines have been shown to activate immune cells and the inflammatory signal transduction of adjacent non-immune cells. This occurs through c-Jun N-terminal kinase (JNK), an inhibitor of nuclear factor kappa B kinase subunit beta (IKKβ)/nuclear factor-kappa B (NF-κB), and other signaling pathways, causing immune cell infiltration and aggravating inflammation (Lee et al., 2014; Shin et al., 2017; Jaiswal et al., 2019).

Obesity causes great harm to the liver and cardiovascular system. The liver is a key site of metabolic homeostasis and its inflammation is closely related to the occurrence and development of obesity. Epidemiological investigations have found that 50%–90% of people with obesity have NAFLD (Divella et al., 2019). In the liver, IKK-β/NF-κB signaling pathways are activated by obesity and a high fat diet (HFD), and higher levels of inflammatory cytokines are released (Dou et al., 2018; Heida et al., 2021). Furthermore, in obesity, the abnormal lipid metabolism and the chronic inflammation can cause direct damage to the vascular endothelium, leading to vascular endothelial dysfunction, inflammation, and a large amount of lipid and immune cell infiltration, resulting in lipid plaques that lead to atherosclerosis (AS), a direct cause of cardiovascular diseases (Kobiyama and Ley, 2018).

Mongolian medicine has a long history of clinical application as part of Traditional Chinese Medicine (TCM). In Mongolian medicine, many classical compound preparations are especially effective in treating chronic metabolic diseases. The Traditional Mongolian Medicine Qiqirigan-8 (MMQ-8) composed by 8 botanical drugs including *K* *aempferia galanga* L. (*Zingiberaceae*; *K*. *galanga* rhizome; Chinese name: Shannai), *Inula helenium* L. (*Compositae*; *I*. *helenium* rhizome et root; Chinese name: Tumuxiang), *Dolomiaea costus* (Falc.) Kasana and A.K. Pandey (*Asteraceae*; *D*. *costus* root; Chinese name: Muxiang), *Rheum palmatum* L. (*Polygonaceae*; *R*. *palmatum* radix et rhizome; Chinese name: Dahuang), *Hippophae rhamnoides* L (*Elaeagnaceae*; *H*. *rhamnoides* fruit; Chinese name: Shaji), *Piper longum* L. (*Piperaceae*; *P*. *longum* fruit; Chinese name: Biba), *Biancaea sappan* (L.) Tod. (*Leguminosae*; *Caesalpinia sappan* heart wood; Chinese name: Sumu), and *Sus scrofa* L. (*Suidae*; *S*. *scrofa* processed feces; Chinese name: Heibingpian). In the clinic, MMQ-8 has great therapeutic effects on obesity, dyslipidemia, hypertension, AS, and coronary heart disease. Numerous studies have demonstrated that the botanical drugs such as *K*. *galanga* L., *H*. *rhamnoides* L., *R*. *palmatum* L., and *P*. *longum* L. have anti-inflammatory, antioxidant, and anti-hyperlipidemia effects (Yang et al., 2016; Tkacz et al., 2019; Arunachalam et al., 2020; Wahyuni et al., 2022). These botanical drugs are rich in terpenoids, flavonoids, alkaloids, phenolic acids, fatty acids, and other substances that have multiple pharmacological effects. Baicalein, boldine, scutellarein, quercetin, and conjugated linoleic acid are all known to optimize lipid metabolism and decrease inflammation by regulating NF-κB, peroxisome proliferator-activated receptor (PPAR), and the phosphatidylinositol 3-kinase (PI3K) signaling pathway (Yang et al., 2018; den Hartigh, 2019; Lin et al., 2019; Yang et al., 2019; Dai et al., 2021). Therefore, we believe that MMQ-8 has a regulatory effect on lipid metabolism and inflammation. However, the active metabolites and pharmacological mechanism of MMQ-8 have not been characterized.

In this study, we first determined the active metabolites of MMQ-8 using ultrahigh-performance liquid chromatograph Q extractive mass spectrometry (UHPLC-QE-MS) and network analysis. Bioinformatics analysis and experimental validation were performed to examine the treatment effects of MMQ-8 on lipid metabolism and inflammation.

## Materials and Methods

### Animal Models and Treatment

Wistar rats (male, 6–8 weeks old, 180 ± 20 g) were purchased from SiPeiFu Co. (Beijing, China). Rats were maintained in a normal environment on a 12-h light/dark cycle (lights on 06:00–18:00 h) with ad libitum access to food and water and were fed a standard diet in the control group. The other groups were fed an HFD (70% normal chow, 10% lard oil, 10% yolk powder, 5% sugar, 4.5% cholesterin, and 0.5% cholate) for 10 months. All animal experiments were permitted by the Institutional Animal Care and Use Committee of Inner Mongolia Medical University.

All rats were randomly divided into six groups (*n* = 8): Control, Model, MMQ-8 (A&B), and Simvastatin (ST) (A&B). The model group was given saline (2 ml) daily for 10 months; the control group had no treatment. The MMQ-8(A) group was given MMQ-8 (312.5 mg/kg) daily for 10 months. The MMQ-8(B) group was given MMQ-8 (312.5 mg/kg) daily for 5 months after 5 months of modeling. The ST(A) group was given Simvastatin tablets (2.08 mg/kg) daily for 10 months. The ST(B) group was given Simvastatin tablets (2.08 mg/kg) daily for 5 months after 5 months of modeling.

Preparing drugs and dose calculations: MMQ-8 botanicals ratio are *K*. *galanga* L. (21062907, Anguo Jiuwang Pharmaceutical Co., Ltd., China) is 207 g; *I*. *helenium* L.(C20071008, Anguo Runde Pharmaceutical Co., Ltd., China) is 66 g; *D*. *costus* (Falc.) Kasana and A.K. Pandey (2106011212, Hebei Liankang Pharmaceutical Co., Ltd., China) is 66 g; *R*. *palmatum* L. (21042603, Anguo Jiuwang Pharmaceutical Co., Ltd., China) is 41 g; *H*. *rhamnoides* L. (C201021612, Anguo Runde Pharmaceutical Co., Ltd., China) is 248 g; *P*. *longum* L. (C790210101, Anguo Ronghua Materia Medica Co., Ltd., China) is 165 g; *B*. *sappan* (L.) Tod (C480201202, Anguo Ronghua Materia Medica Co., Ltd., China) is 124 g; *S*. *scrofa* L. (20022701, Inner Mongolia Muxin Pharmaceutical Co., Ltd., China) is 83 g. The decoction pieces of these botanicals were mixed according to the ratio. Then, the mixture was smashed using a pulverizer and filtered with a 70-mesh sieve. If the decoction pieces could not be filtered, we continued to pulverize and sieve the mixture until all pass through the filter. Finally, it was carefully dried and stored in a cool, dry place. Clinical administration requires an MMQ-8 dose of 3 g each time with an adult body weight of 60 kg, which means the dose was 50 mg/kg body weight. The equivalent dose ratio of human to rat based on body surface area is 6.25. Therefore, the MMQ-8 dose given to rats per kg of body weight was 312.5 mg (Formula: MMQ-8 50 mg/kg*6.25 = 312.5 mg/kg). In addition, the simvastatin dose for hyperlipidemia is 20 mg, which means the adult dose was 0.33 mg/kg body weight. Thus, the dose given to rats per kg of body weight was 2.08 mg (formula: 0.33 mg/kg*6.25 = 2.08 mg/kg). Finally, after calculating the dose for each rat, the drugs were dissolved in 2 ml of pure water. All the treatments were administered orally once a day at a fixed time.

### Cell Culture and Treatment

Mouse T lymphocyte cells (CTLL-2; BeNa Culture Collection, China) were used in this study and routinely cultured in 1640 (Gibco, United States). CTLL-2 cells were supplemented with 10% fetal bovine serum (FBS; Biological Industries, United States) and 1% penicillin/streptomycin (Sigma, United States) and cultured at 37°C in a humidified atmosphere containing 5% CO_2_. The CTLL-2 cell treatment was supplemented with 1% MMQ-8 rat serum and normal rat serum, respectively, and cultured for 24 h at 37°C in a humidified atmosphere containing 5% CO_2_.

### CCK-8 Assay

CTLL-2 cells were cultured in 96-well plates. Then cells were incubated with different concentrations of MMQ-8 serum: 0, 1, 2, 3, 4, 5, 6, 7, 8, 9, and 10%. After 24 h, CCK-8 was added and cultured for 4 h. Then absorption values were calculated using a microplate reader at 450 nm. Inhibition rate = [(Ac-As)/(Ac-Ab)] 100% (As: including cell, medium, CCK-8 and MMQ-8 serum; Ac: including cell, medium and CCK-8; Ab: including medium and CCK-8).

### UHPLC-QE-MS

Metabolite extraction: 100 mg of TCM sample was added to 500 μl of the extracted solution containing 1 μg/ml of internal standard. The samples were then homogenized at 40 Hz for 4 min and sonicated for 1 h in an ice-water bath. After resting for 1 h at −20°C, the samples were centrifuged at 12,000 rpm [RCF = 13,800 (×g), R = 8.6 cm] for 15 min at 4°C. Finally, 300 μl of the supernatant was carefully filtered through a 0.22 μm microporous membrane and placed in a fresh 2 ml tube for LC-MS/MS analysis.

LC-MS/MS conditions: LC-MS/MS analysis was performed on an Agilent ultra-high performance liquid chromatography 1290 UPLC system with a Waters UPLC BEH C18 column (1.7 μm 2.1*100 mm). The flow rate was set at 0.4 ml/min and the sample injection volume was set at 3 μl. The mobile phase consisted of 0.1% formic acid in water (A) and 0.1% formic acid in acetonitrile (B). The multi-step linear elution gradient program was as follows: 0–3.5 min, 95%–85% A; 3.5–6 min, 85%–70% A; 6–6.5 min, 70%–70% A; 6.5–12 min, 70%–30% A; 12–12.5 min, 30%–30% A; 12.5–18 min, 30%–0% A; 18–25 min, 0%–0% A; 25–26 min, 0%–95% A; 26–30 min, 95%–95% A.

An AQ Exactive Focus mass spectrometer coupled with Xcalibur software was used to obtain the MS and MS/MS data based on the IDA acquisition mode. During each acquisition cycle, the mass range was between 100 and 1,500. The top three of every cycle were screened and the corresponding MS/MS data were acquired. Specifications were as follows sheath gas flow rate: 45 Arb; Aux gas flow rate: 15 Arb; Capillary temperature: 400°C; Full ms resolution: 70,000; MS/MS resolution: 17,500; Collision energy: 15/30/45 in NCE mode; Spray Voltage: 4.0 kV (positive) or −3.6 kV (negative).

### Network Analysis

The chemical compositions of MMQ-8 detected by UHPLC-QE-MS were screened according to the Percent Human Oral Absorption rate (≥30%) and drug likeness (DL) (≥0.185). Then chemical component targets of MMQ-8 were obtained from the CHEMBL database (https://www.ebi.ac.uk/chembl/). In the Gene Cards Database (https://www.genecards.org) and the Therapeutic Target Database (TTD) (https://db.idrblab.net/ttd/), the keywords “inflammation,” “obesity,” and “lipid metabolism” were input to obtain relevant target genes. Then the UniProtKB search function of the UniProt database (http://www.uniprot.org/) was used to standardize the names of those predicted target genes. Finally, Venn analysis was performed on MMQ-8 target genes, obesity-related genes, and inflammation-related genes to obtain common target genes. This took place using GO (http://www.geneontology.org/) and KEGG pathway (www.kegg.jp/kegg/pathway.html) analysis.

### Serum Sampling and ELISA Assay

To detect the activities of serum blood lipids, liver enzymes, and inflammatory factors, the levels of triglyceride (TG), total cholesterol (TC), low-density lipoprotein (LDL-C), high-density lipoprotein (HDL-C), alanine aminotransferase (ALT), and aspartate aminotransferase (AST) were assessed using commercially available diagnostic kits (Nan Jing Jian Cheng Bio Co., Ltd., China). The serum levels of inflammatory factors TNF-α, inducible nitric oxide synthase (INOS), IL-6, and IL-10 were detected using an enzyme-linked immunosorbent assay (ELISA) kit (Jiang Su Mei Biao Bio Co., Ltd., China). While strictly following the ELISA kit instructions, detection was performed using a Multi-Plate Reader (Biotek Synergy, United States) with Gen5 software. Serum TNF-α, IL-1β, IL-6, and IL-10 contents were calculated according to the standard curve.

### Histology and Immunohistochemistry

Liver tissue sections from the same part of the liver lobe were cut, fixed in 4% buffered formalin at room temperature for 24 h, embedded in paraffin, and sliced into 4-μm sections. Sections were stained with hematoxylin and eosin (H&E). Histopathological changes were checked under a microscope. Furthermore, we assigned NAFLD activity scores (NAS) to liver H&E images. The scoring criteria are shown in Table 1. The arcus aorta was stained with H&E and methods were the same as those for liver tissues.

**TABLE 1:** The scoring criteria of NAS.

Pathological characteristic	Assessment	Score
Hepatocyte ballooning	none	0
	a little	1
	a lot	2
Lobular inflammation	none	0
(inflammatory foci/200x	<2	1
field of view)	2–4	2
	>4	3
Steatosis	<5%	0
	5%–33%	1
	33%–66%	2
	>66%	3
Pathological diagnosis		Total score

Histological visualization of lipid deposition in rat hepatic tissues was performed using oil red O staining. Briefly, frozen sections of rat liver tissue were first incubated in distilled water for 2 min, then 60% propylene glycol for 2 min, and finally in oil red O solution for 5 min. Then the sections were soaked in 60% propylene glycol until the interstitial tissue was colorless. After rinsing for 2 min with distilled water, the slides were incubated at 37°C with hematoxylin for 1 min and mounted with gelatin mounting medium.

Immunohistochemistry was performed on the arcus aorta sections. The sections were immersed in distilled water at room temperature for 25 min. The sections were blocked with 3% rabbit serum and then incubated overnight at 4°C with primary antibodies: anti-CD68 (ab283654, Abcam) and anti-Mannose receptor CD206 (ab64693, Abcam). The sections were washed followed by incubation with corresponding goat anti-rabbit IgG H&L (HRP) (ab6721, Abcam) for 50 min at room temperature. They were then immersed in PBS for 5 min and stained with DAB and hematoxylin. The histopathological changes were checked under a microscope.

### RNA Sequencing

Muscle tissue was removed from the femur and tibia and rinsed with PBS. Tissue scissors were used to cut off both ends of the bone, and marrow cells were flushed out using a syringe containing PBS. The cell suspension was centrifuged at 1000 rpm for 5 min to collect bone marrow cells. Then red blood cell lysis buffer was added in moderation to the bone marrow cells to remove all red blood cells. They were then cleaned with PBS and an appropriate amount of TRIZOL UP was added. Cells were then frozen at −80°C after complete mixing. Finally, the total RNA was extracted to synthesize cDNA sequenced using an Illumina platform and the information was analyzed. The genes that displayed a >1.5-fold change in expression—and based on at least two pairwise comparisons with the same trend—were selected for further examination in the test phase (*p* ˂ 0.01).

### Quantitative Real-Time PCR

Total RNA was isolated from cultured cells using RNAiso Plus reagent (TAKARA, Japan) according to the manufacturer’s instructions. The total RNA was directly used for RT-qPCR by Trans Script Green One-Step qRT-PCR Super Mix reagent (Trans Gen Biotech, China) following the manufacturer’s protocol on an ABI Prism7500 instrument (ABI, United States). Details of the mRNA—specific primers for GAPDH, TNF-α, interferon regulatory factor 1(IRF1), T-bet, signal transducer and activator of transcription (STAT)1, STAT3, STAT4, STAT6, GATA binding protein 3 (GATA3), IL-10, RORγt, nuclear factor-kappa B inhibitor alpha (IκBα), and IL-17A are shown in Table 2. The relative mRNA levels were determined using the comparative Ct method with GAPDH as the reference gene, and the formula 2^−ΔΔCt^.

**TABLE 2:** Primers used in this study for RT-PCR.

Gene	Forward Primer (5’→3′)	Reverse Primer (5’→3′)
GAPDH	GCG​ACT​TCA​ACA​GCA​ACT​CCC	CAC​CCT​GTT​GCT​GTA​GCC​GTA
TNF-α	AGT​TCT​ATG​GCC​CAG​ACC​CTC	TGT​CTT​TGA​GAT​CCA​TGC​CGT​T
IRF1	ACA​TAA​CTC​CAG​CAC​TGT​CAC​C	TTC​CCT​TCC​TCA​TCC​TCG​TCT
T-bet	GTA​TCC​TGT​TCC​CAG​CCG​TTT	TCA​TAA​CTG​TGT​TCC​CGA​GGT
STAT1	TCA​CAG​TGG​TTC​GAG​CTT​CAG	GCA​AAC​GAG​ACA​TCA​TAG​GCA
STAT3	CAA​TAC​CAT​TGA​CCT​GCC​GAT	GAG​CGA​CTC​AAA​CTG​CCC​T
STAT4	TGG​CAA​CAA​TTC​TGC​TTC​AAA​AC	GAG​GTC​CCT​GGA​TAG​GCA​TGT
STAT6	CTC​TGT​GGG​GCC​TAA​TTT​CCA	CAT​CTG​AAC​CGA​CCA​GGA​ACT
GATA3	CTC​GGC​CAT​TCG​TAC​ATG​GAA	GGA​TAC​CTC​TGC​ACC​GTA​GC
IL-10	GCT​CTT​ACT​GAC​TGG​CAT​GAG	CGC​AGC​TCT​AGG​AGC​ATG​TG
RORγt	GAC​CCA​CAC​CTC​ACA​AAT​TGA	AGT​AGG​CCA​CAT​TAC​ACT​GCT
IκBα	TGA​AGG​ACG​AGG​AGT​ACG​AGC	TTC​GTG​GAT​GAT​TGC​CAA​GTG
IL-17A	TTT​AAC​TCC​CTT​GGC​GCA​AAA	CTT​TCC​CTC​CGC​ATT​GAC​AC

### Western Blot

The vascular and liver tissues that were frozen at −80°C were removed from the refrigerator and rinsed twice with PBS. Tissues were then cut into small pieces and put into a glass grinder with an appropriate amount of TPEB to be ground for half an hour on ice. Protein samples were obtained by centrifugation at 12,000 rpm for 10 min at 4°C. Protein concentrations were determined by BCA protein assays (Thermo Scientific, United States), and approximately 25 μg of total protein was used for SDS-PAGE through a 12% running and 5% stacking gel. Samples were transferred to a nitrocellulose transfer membrane (66485, Pall) using the wet transfer method. Membranes were blocked with 5% non-fat dry milk and then incubated overnight at 4°C with primary antibodies and corresponding secondary antibodies: anti-p38 (1:1000; 8690, CST), anti-IκBα (1:1000; 4814, CST), anti-SAPK/JNK (1:1000; 9252, CST), anti-ERK1/2 (1:1000; 4695, CST), anti Phospho-p38 (1:1000; 4511, CST), anti Phospho-IκBα (1:1000; 2859, CST), anti Phospho-SAPK/JNK (1:1000; 4668, CST), anti Phospho-ERK1/2 (1:1000; 4370, CST), GAPDH (1:1000; 5174, CST), and anti β-Actin (1:1000; 4970, CST). Membranes were washed, followed by incubation with corresponding IRDye^®^ 800LT goat anti-rabbit (1:20,000; 5151, CST) and anti-mouse (1:20,000; 5257, CST) for 1 h at approximately 25°C. The primary antibody and corresponding secondary antibody were evaluated separately. After scanning, successive secondary rounds of incubation were performed for each membrane using seven different antibodies recognizing seven different proteins. Detection and quantification were performed using an Infrared Imaging System (Odyssey; LI-COR Biosciences). Band intensities were determined using the median background method. The mitogen-activated protein kinase (p38), IκBα, extracellular regulated kinase 1/2(ERK1/2), stress-activated protein kinase/c-Jun N-terminal kinase (SAPK/JNK), Phospho-p38, Phospho-IκBα, Phospho-SAPK/JNK, and Phospho-ERK1/2 levels were normalized to β-actin or GAPDH.

### Statistics

All data are represented as mean ± SEM. Significance was tested using one-way ANOVA, followed by Dunnett’s multiple comparisons, when appropriate (GraphPad Prism 7, United States). Results with *p*-values < 0.05 were considered statistically significant.

## Results

### MMQ-8 Chemical Composition

**FIGURE 1:**
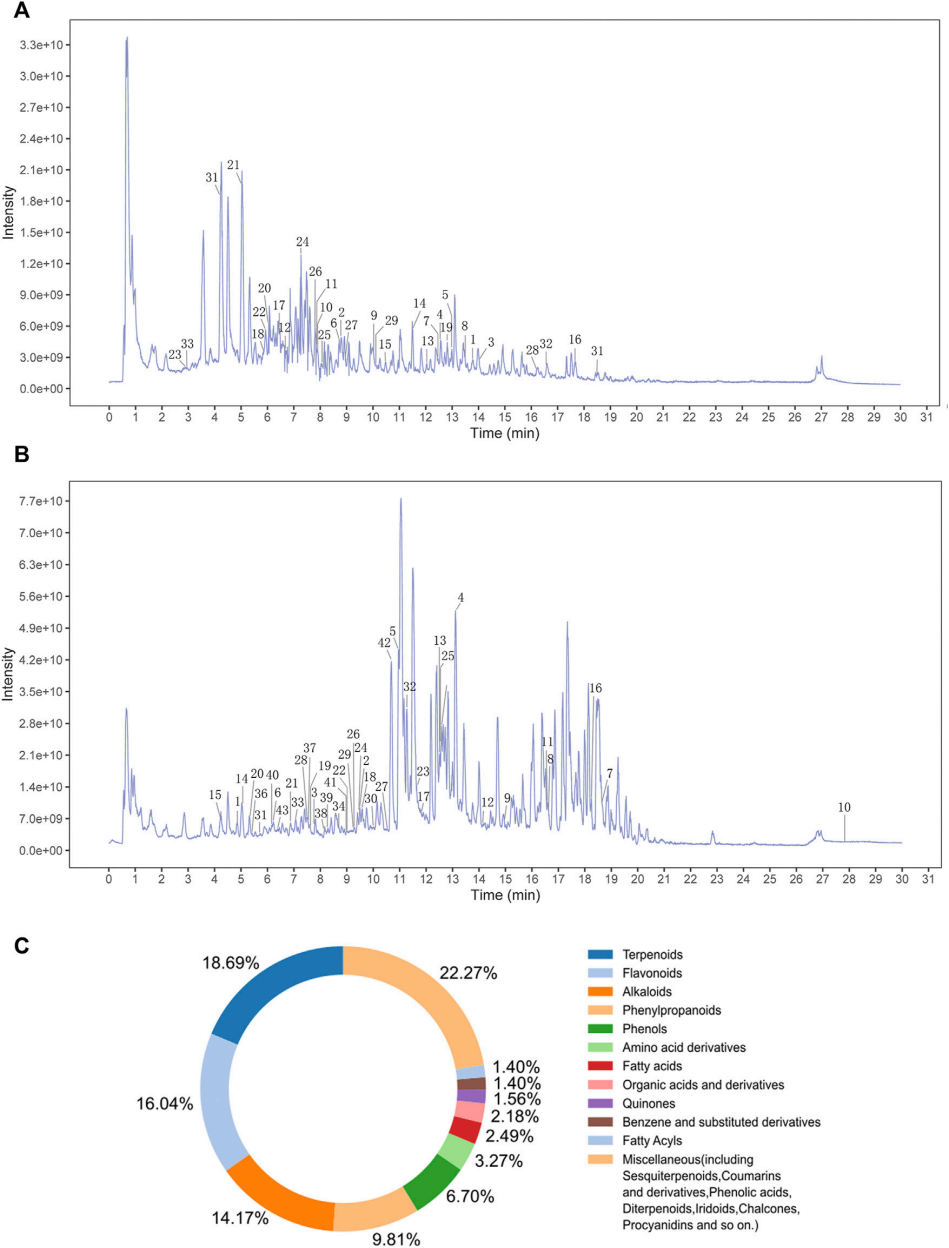
An overview of MMQ-8 chemical components was detected by UHPLC-QE-MS assay. **(A)** The fingerprint of MMQ-8 total chemical components in NEG electrospray ionization modes. We marked 33 active metabolites screened according to the Percent Human Oral Absorption and drug likeness values **(B)** The fingerprint of MMQ-8 total chemical components detected in POS electrospray ionization modes. We marked 43 active metabolites screened according to the Percent Human Oral Absorption and drug likeness values. **(C)** In total, 642 chemical components of MMQ-8 were obtained, among which flavonoids, terpenoids, phenylpropanoids, phenols, and alkaloids were the most abundant.

TCM is characterized by multi-component, multi-target integration and regulation. Thus, it is important for research to determine the active metabolites of drugs (Zhang et al., 2020). In this study, we set out to use the UHPLC-QE-MS assay to determine the chemical composition of MMQ-8. As shown in Figures 1A,B, we obtained 303 chemical components in the negative (NEG) electrospray ionization modes of UHPLC-QE-MS, and 404 in the positive (POS) electrospray ionization modes. A total of 642 chemical components were obtained that contained 51 types of compounds and the content of flavonoids, terpenoids, phenylpropanoids, phenols, and alkaloids were the largest of all the chemical components of MMQ-8 (Figure 1C). These compounds are widely present in plants and most have anti-inflammatory, antioxidant, anti-cancer, antibacterial, liver protection, cardiovascular protection, and other pharmacological effects (Zhang et al., 2019; Liao et al., 2020; Yuan et al., 2021). These results suggest that MMQ-8 has multiple pharmacological effects. We performed a network analysis of MMQ-8 chemical components to confirm the medicinal values.

### The Relationship Between MMQ-8 Metabolites, Obesity, and Inflammation

**FIGURE 2:**
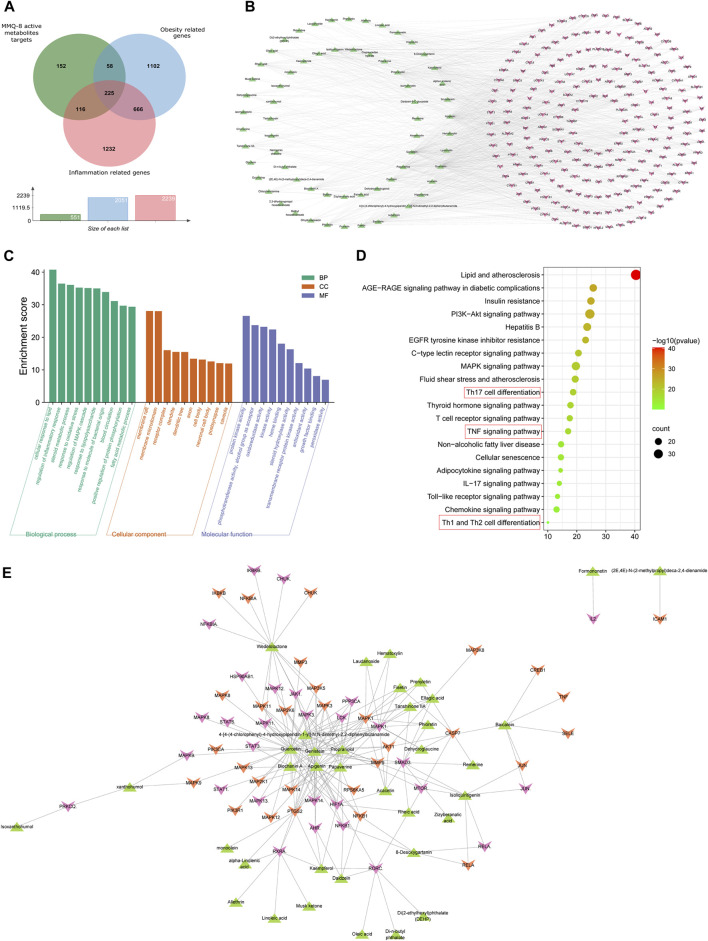
continued

The therapeutic effects of TCM involve complex processes that operate through the synergistic action of various compounds on different targets (Zhao et al., 2021). To explore the pharmacological action of MMQ-8, we carried out network analysis based on the MMQ-8 components detected in the UHPLC-QE-MS assay. We obtained a total of 69 potentially active metabolites of MMQ-8 that were screened according to the Percent Human Oral Absorption (≥30%) and drug likeness values (≥0.185). As shown in Figures 1A,B, we marked the potentially active metabolites in fingerprints and more details are shown in Tables 3, 4. In the ChEMBL database, we identified 551 target genes relating to the active metabolites of MMQ-8. There were 2,061 obesity-related genes and 2,239 inflammation-related genes obtained from TTD. A total of 225 common target genes were identified among MMQ-8 target genes, obesity related genes, and inflammation related genes (Figure 2A). These 225 common target genes were regulated by the 65 active metabolites of MMQ-8 (Figure 2B). GO and KEGG enrichment analyses were then performed on these 225 target genes. GO analysis indicated that enrichment of oxidative, inflammation, and lipid metabolism was highly significant (Figure 2C). In KEGG enrichment analysis, enrichment of lipid, AS, T cell receptor signaling pathway, TNF signaling pathway, NAFLD, and T-helper (Th) 17 cell differentiation signaling pathways were the most significant (Figure 2D). The TNF signaling pathway is a classic inflammatory signal transduction pathway, which is very large and includes many downstream signaling pathways, such as NF-κB, mitogen-activated protein kinase (MAPK), and PI3K (Kalliolias and Ivashkiv, 2016). In addition, we have observed that MMQ-8 has significantly inhibiting effects on the inflammatory signaling pathway TNF and Th cell differentiation related signaling pathway *in vivo* and *in vitro*. Therefore, we performed further analysis on TNF signaling pathway, Th1 and 2 cell differentiation, and Th17 cell differentiation signaling pathways and obtained 37 active metabolites of MMQ-8 that directly participate in regulating these signaling pathways, as shown in Figure 2E. These findings suggest that MMQ-8 has regulatory effects on lipid metabolism and inflammation.

**TABLE 3:** Thirty-three MMQ-8 active metabolites have been detected in NEG electrospray ionization modes of UHPLC-QE-MS assay and screened by the Percent Human Oral Absorption and drug-likeness values.

Peak no.	Chemical components name	InChIKey	Formula	Class	rtmed	MMQ-.8
1	Linoleic acid	OYHQOLUKZRVURQ-HZJYTTRNSA-N	C18H32O2	Fatty Acyls	13.86	3908299.68
2	Scutellarein	JVXZRQGOGOXCEC-UHFFFAOYSA-N	C15H10O6	Flavonoids	8.89	42271653.76
3	Ginkgolic acid C17-1	MBYNDKVOZOAOIS-FPLPWBNLSA-N	C24H38O3	Phenolic acids	14.27	360133.87
4	Acacetin	DANYIYRPLHHOCZ-UHFFFAOYSA-N	C16H12O5	Flavonoids	12.56	6993462.76
5	Daidzein	ZQSIJRDFPHDXIC-UHFFFAOYSA-N	C15H10O4	Flavonoids	13.04	28079447.48
6	Genistein	TZBJGXHYKVUXJN-UHFFFAOYSA-N	C15H10O5	Flavonoids	8.71	23184570.51
7	Methyl hexadecanoate	FLIACVVOZYBSBS-UHFFFAOYSA-N	C17H34O2	Fatty Acyls	12.54	228205245.45
8	Palmitic acid	IPCSVZSSVZVIGE-UHFFFAOYSA-N	C16H32O2	Fatty acids	13.48	2829838.42
9	Aloeemodin	YDQWDHRMZQUTBA-UHFFFAOYSA-N	C15H10O5	Quinones	10.12	54173807.97
10	Quercetin	REFJWTPEDVJJIY-UHFFFAOYSA-N	C15H10O7	Flavonoids	7.94	310447894.57
11	Pinocembrin	URFCJEUYXNAHFI-UHFFFAOYSA-N	C15H12O4	Flavonoids	7.92	11150537.32
12	isoimperatorin	IGWDEVSBEKYORK-UHFFFAOYSA-N	C16H14O4	Coumarins and derivatives	6.71	18470601.53
13	Fisetin	XHEFDIBZLJXQHF-UHFFFAOYSA-N	C15H10O6	Flavonoids	12.14	5330818.86
14	Prenyletin	AWEFUQDNSBBNCR-UHFFFAOYSA-N	C14H14O4	Phenylpropanoids	11.60	31376050.12
15	Rheic acid	FCDLCPWAQCPTKC-UHFFFAOYSA-N	C15H8O6	Quinones	10.48	506969375.75
16	Oleic acid	ZQPPMHVWECSIRJ-KTKRTIGZSA-N	C18H34O2	Fatty acids	17.69	107660221.94
17	Kaempferol-3-O-rutinoside	RTATXGUCZHCSNG-QHWHWDPRSA-N	C27H30O15	Flavonoids	6.39	297808697.00
18	Ellagic acid	AFSDNFLWKVMVRB-UHFFFAOYSA-N	C14H6O8	Phenols	5.88	316699609.73
19	Di-n-butyl phthalate	DOIRQSBPFJWKBE-UHFFFAOYSA-N	C16H22O4	Organic acids and derivatives	12.74	15817642.96
20	Biochanin A	WUADCCWRTIWANL-UHFFFAOYSA-N	C16H12O5	Flavonoids	5.98	1370100199.65
21	Oxypeucedan hydrate	PEWFWDOPJISUOK-UHFFFAOYSA-N	C16H16O6	Phenylpropanoids	5.05	377495449.04
22	Kaempferide	SQFSKOYWJBQGKQ-UHFFFAOYSA-N	C16H12O6	Flavonoids	5.93	45821248.89
23	Hematoxylin	WZUVPPKBWHMQCE-UHFFFAOYSA-N	C16H14O6	Flavonoids	2.88	22993795.20
24	Isoginkgetin	HUOOMAOYXQFIDQ-UHFFFAOYSA-N	C32H22O10	Flavonoids	7.28	21679037.51
25	Phloretin	VGEREEWJJVICBM-UHFFFAOYSA-N	C15H14O5	Flavonoids	8.31	7418001.46
26	Wedelolactone	XQDCKJKKMFWXGB-UHFFFAOYSA-N	C16H10O7	Phenylpropanoids	7.80	28242363.39
27	Isorhamnetin	IZQSVPBOUDKVDZ-UHFFFAOYSA-N	C16H12O7	Flavonoids	9.05	379239800.95
28	Isoxanthohumol	YKGCBLWILMDSAV-UHFFFAOYSA-N	C21H22O5	Chalcones	16.24	112424264.07
29	4-[2-(2,6-dimethoxy-4-prop-2-enylphenoxy)-1-hydroxypropyl]-2-methoxyphenol	ULZFTGWWPHYLGI-UHFFFAOYSA-N	C21H26O6	Miscellaneous	10.13	18399869.54
30	Hispidulin	IHFBPDAQLQOCBX-UHFFFAOYSA-N	C16H12O6	Flavonoids	4.20	136805092.85
31	Baicalein	FXNFHKRTJBSTCS-UHFFFAOYSA-N	C15H10O5	Flavonoids	18.51	4909421.35
32	8-Desoxygartanin	GVQOVMKBYJKZSY-UHFFFAOYSA-N	C23H24O5	Xanthones	16.62	127605764.90
33	Naringenin chalcone	YQHMWTPYORBCMF-ZZXKWVIFSA-N	C15H12O5	Flavonoids	2.98	12120512.80

**TABLE 4:** Forty-three MMQ-8 active metabolites have been detected in POS electrospray ionization modes of UHPLC-QE-MS assay and screened by the Percent Human Oral Absorption and drug-likeness values.

Peak no.	Chemical components name	InChIKey	Formula	Class	rtmed	MMQ-.8
1	Dehydroglaucine	RZUHGAKUNBFQJS-UHFFFAOYSA-N	C21H23NO4	Alkaloids	4.90	338700.94
2	Prunetin	KQMVAGISDHMXJJ-UHFFFAOYSA-N	C16H12O5	Flavonoids	9.56	22018287.57
3	Biochanin A	WUADCCWRTIWANL-UHFFFAOYSA-N	C16H12O5	Flavonoids	7.74	554287545.01
4	alpha-Linolenic acid	DTOSIQBPPRVQHS-PDBXOOCHSA-N	C18H30O2	Fatty Acyls	13.11	750206063.91
5	Piperanine	QHWOFMXDKFORMO-XVNBXDOJSA-N	C17H21NO3	Alkaloids	10.96	28890056271.05
6	Kaempferol	IYRMWMYZSQPJKC-UHFFFAOYSA-N	C15H10O6	Flavonoids	6.30	81334291.04
7	Rotundine	AEQDJSLRWYMAQI-UHFFFAOYSA-N	C21H25NO4	Alkaloids	18.68	43512140.81
8	2,3-dihydroxypropyl hexadecanoate	QHZLMUACJMDIAE-UHFFFAOYSA-N	C19H38O4	Miscellaneous	16.67	153597508.15
9	monoolein	RZRNAYUHWVFMIP-KTKRTIGZSA-N	C21H40O4	Ester	14.95	85289125.39
10	Di (2-ethylhexyl)phthalate (DEHP)	BJQHLKABXJIVAM-UHFFFAOYSA-N	C24H38O4	Miscellaneous	27.88	1853021.65
11	Linoleic acid	OYHQOLUKZRVURQ-HZJYTTRNSA-N	C18H32O2	Fatty Acyls	16.62	134004328.85
12	Tanshinone IIA	HYXITZLLTYIPOF-UHFFFAOYSA-N	C19H18O3	Diterpenoids	14.13	35506306.90
13	(2E,4E)-N-(2-methylpropyl)deca-2,4-dienamide	MAGQQZHFHJDIRE-BNFZFUHLSA-N	C14H25NO	Miscellaneous	12.50	9498955663.63
14	4-[4-(4-chlorophenyl)-4-hydroxypiperidin-1-yl]-N,N-dimethyl-2,2-diphenylbutanamide	RDOIQAHITMMDAJ-UHFFFAOYSA-N	C29H33ClN2O2	Alkaloids	5.08	595202.27
15	Glycitein	DXYUAIFZCFRPTH-UHFFFAOYSA-N	C16H12O5	Flavonoids	4.25	61336663.26
16	Laudanoside	KGPAYJZAMGEDIQ-UHFFFAOYSA-N	C21H27NO4	Alkaloids	18.36	814876194.31
17	Baicalein	FXNFHKRTJBSTCS-UHFFFAOYSA-N	C15H10O5	Flavonoids	11.80	18364679.77
18	Remerine	JCTYWRARKVGOBK-UHFFFAOYSA-N	C18H17NO2	Alkaloids	9.57	20462783.36
19	suberosin	RSZDAYHEZSRVHS-UHFFFAOYSA-N	C15H16O3	Phenylpropanoids	7.69	40617001.09
20	Boldine	LZJRNLRASBVRRX-UHFFFAOYSA-N	C19H21NO4	Alkaloids	5.40	130502155.57
21	Tectochrysin	IRZVHDLBAYNPCT-UHFFFAOYSA-N	C16H12O4	Flavonoids	6.90	899216497.98
22	Higenamine	WZRCQWQRFZITDX-UHFFFAOYSA-N	C16H17NO3	Alkaloids	9.03	20164741.63
23	Cinchonine	KMPWYEUPVWOPIM-UHFFFAOYSA-N	C19H22N2O	Alkaloids	11.65	16887401.82
24	Musk ketone	WXCMHFPAUCOJIG-UHFFFAOYSA-N	C14H18N2O5	Alkaloids	9.49	13299260.88
25	Zizyberanalic acid	SLWJVQQNDGLXTK-UHFFFAOYSA-N	C30H46O4	Terpenoids	12.59	59303945.96
26	Propranolol	AQHHHDLHHXJYJD-UHFFFAOYSA-N	C16H21NO2	Benzenoids	9.38	74955671.48
27	xanthohumol	ORXQGKIUCDPEAJ-YRNVUSSQSA-N	C21H22O5	Flavonoids	10.07	83091714.83
28	Naringenin chalcone	YQHMWTPYORBCMF-ZZXKWVIFSA-N	C15H12O5	Flavonoids	7.58	30710288.38
29	Formononetin	HKQYGTCOTHHOMP-UHFFFAOYSA-N	C16H12O4	Flavonoids	9.29	31752046.23
30	Papaverine	XQYZDYMELSJDRZ-UHFFFAOYSA-N	C20H21NO4	Alkaloids	9.99	12503204.08
31	Chlorpheniramine	SOYKEARSMXGVTM-UHFFFAOYSA-N	C16H19ClN2	Alkaloids	5.71	5190412.73
32	Allethrin	ZCVAOQKBXKSDMS-UHFFFAOYSA-N	C19H26O3	Terpenoids	11.34	133359654.70
33	Daidzein-8-C-glucoside	HKEAFJYKMMKDOR-UHFFFAOYSA-N	C21H20O9	Flavonoids	7.05	16799822.33
34	Apigenin	KZNIFHPLKGYRTM-UHFFFAOYSA-N	C15H10O5	Flavonoids	8.73	22348134.67
35	2-(8-hydroxy-4a,8-dimethyl-1,2,3,4,5,6,7,8a-octahydronaphthalen-2-yl)prop-2-enoic acid	FXKCXGBBUBCRPU-UHFFFAOYSA-N	C15H24O3	Terpenoids	12.61	103129070.76
36	Wedelolactone	XQDCKJKKMFWXGB-UHFFFAOYSA-N	C16H10O7	Phenylpropanoids	5.44	34482940.62
37	Isoliquiritigenin	DXDRHHKMWQZJHT-FPYGCLRLSA-N	C15H12O4	Flavonoids	7.61	58007436.18
38	Lysionotin	KRFBMPVGAYGGJE-UHFFFAOYSA-N	C18H16O7	Flavonoids	8.15	9912440.23
39	Dihydrocapsaicin	XJQPQKLURWNAAH-UHFFFAOYSA-N	C18H29NO3	Alkaloids	8.36	95100671.72
40	Daidzein	ZQSIJRDFPHDXIC-UHFFFAOYSA-N	C15H10O4	Flavonoids	6.14	27867169.51
41	Dubinidine	NETGEQWGGLFVRL-UHFFFAOYSA-N	C15H17NO4	Alkaloids	8.98	193406036.72
42	Dehydrodiisoeugenol	ITDOFWOJEDZPCF-UHFFFAOYSA-N	C20H22O4	Lignans	10.77	40344890.10
43	Isorhamnetin	IZQSVPBOUDKVDZ-UHFFFAOYSA-N	C16H12O7	Flavonoids	6.40	141579166.87

### MMQ-8 Optimizes Lipid Metabolism

Based on the clinical effects, components and network analysis results of MMQ-8, we decided to establish obesity rat models through HFD and verify the effects of MMQ-8 on lipid metabolism and inflammation. HFD is an important factor that causes obesity. Long-time exposure to an HFD increases the amount of adipose tissue in humans, leading to obesity, weight gain, and steatosis of other tissues and organs. Moreover, studies have confirmed that expansion and anoxia of adipose tissue cause an inflammatory response that is a systemic chronic inflammation closely related to insulin resistance, T2D, AS, NAFLD, and some cancers (González-Muniesa et al., 2017).

**FIGURE 3:**
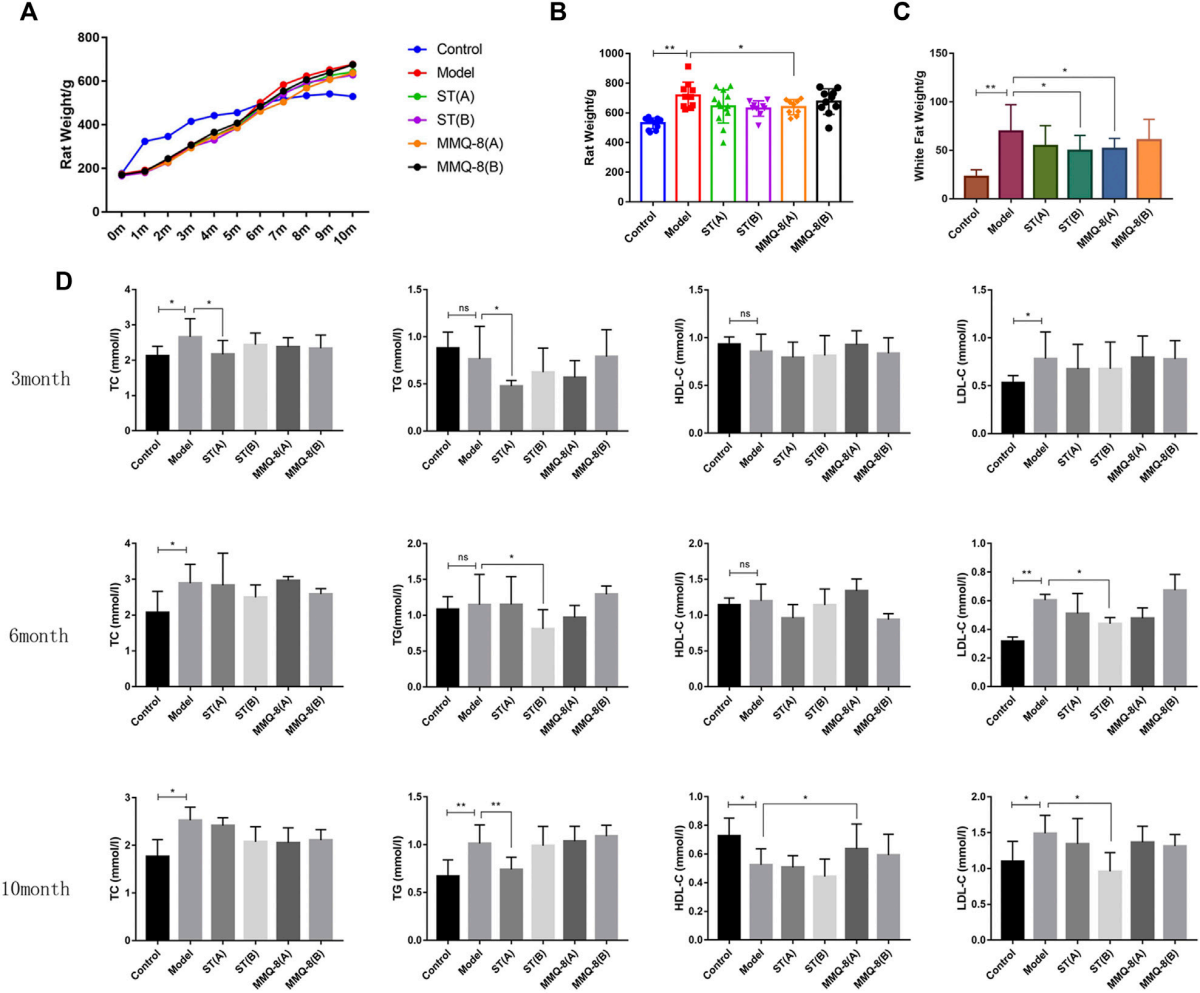
MMQ-8 optimizes lipid metabolism in obesity. **(A)** Statistics of body weight in each month. **(B)** Results of body weight in the 10th month. **(C)** White adipose tissue weight, which includes adipose tissue above the epididymides and around the kidneys. MMQ-8 treatment significantly decreased body weight and white adipose tissue weight. **(D)** Levels of serum lipids measured every 3 months. Serum levels of TC, TG, and LDL-C were decreased and the serum levels of HDL-C were increased after MMQ-8 treatment in obese rats with hyperlipidemia. Data are presented as means ± SD, n = 8, *p < 0.05, **p < 0.01.

We observed that MMQ-8 could optimize lipid metabolism. As shown in Figures 3A–C, compared with the control group, the body weight and white adipose tissue weight of rats in the model group were significantly increased. In the MMQ-8(A) group, the body weight and white adipose tissue weight were significantly decreased compared with the model group. These weights were also reduced in other treatment groups but the difference was not statistically significant compared with the model group.

In addition, blood lipid levels are an important target for observing lipid metabolism. Lipids exist in the blood in the form of TG, cholesterol, and lipoids binding with VLDL, LDL, and HDL. Blood lipid levels change with the absorption of lipids (Yuan et al., 2019). In this study, we tested serum lipid levels three times during the modeling process. As shown in Figure 3D, compared with the control group, TC, TG, and LDL-C levels in the model group were significantly increased, and HDL-C content was significantly decreased in the 10th month. In comparisons between the treatment group and model group, simvastatin significantly down-regulated the levels of TC, TG, and LDL-C, but did not increase HDL-C level. In contrast, MMQ-8 only increased the level of HDL-C. These results suggest that MMQ-8 optimizes lipid metabolism in obese rats.

### MMQ-8 Improved Liver Function and Reduced Hepatic Steatosis and Inflammation

**FIGURE 4:**
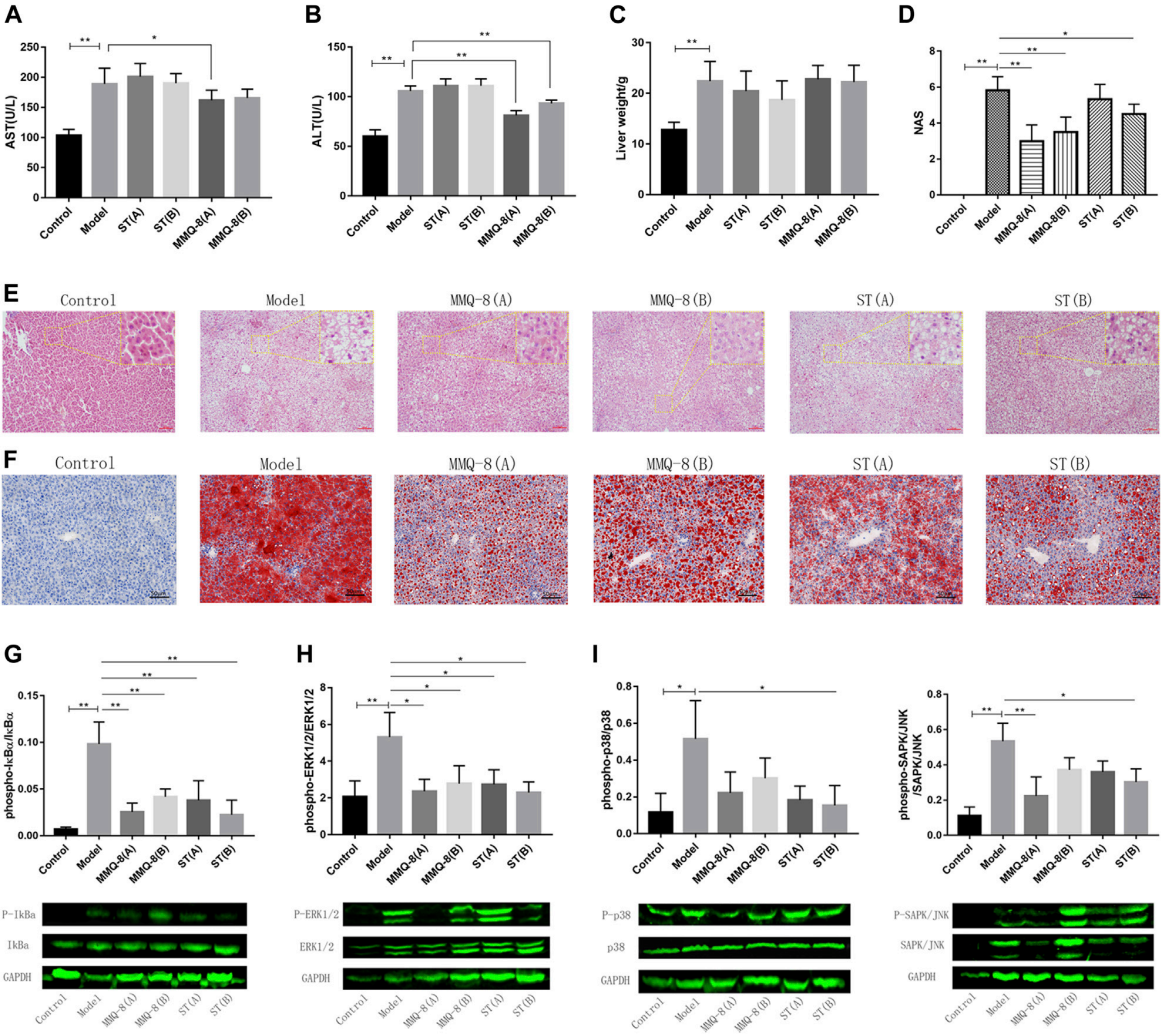
MMQ-8 significantly reduced hepatic steatosis and improved liver function in obese rats fed an HFD. **(A)** Levels of serum hepatic functional marker AST. **(B)** Levels of serum hepatic functional marker ALT. Serum hepatic functional markers were reversed by MMQ-8 treatment. This suggested that MMQ was safe and did not cause liver toxicity. **(C)** Liver wet weights. **(D)** NAS of liver H&E staining images. **(E)** Representative H&E staining of liver (*n* = 6). **(F)** Representative Oil red O staining of liver (*n* = 6). MMQ-8 treatment significantly reduced hepatic steatosis. **(G)** MMQ-8 treatment decreased the phosphorylation levels of IκBα in liver tissue **(H)** MMQ-8 treatment decreased the phosphorylation levels of ERK1/2 in liver tissue. **(I)** MMQ-8 treatment decreased the phosphorylation levels of p38 in liver tissue. **(J)** MMQ-8 treatment decreased the phosphorylation levels of SAPK/JNK in liver tissue. MMQ-8 reduced liver protein phosphorylation levels of the TNF signaling pathway in obese rats. Data are presented as means ± SD, *n* = 8, *p < 0.05, **p < 0.01.

The liver is a key site for lipid metabolism. Lipids accumulate in hepatocytes when the cellular input of fatty acids, *via* either ingestion or synthesis, surpasses fatty acid output, *via* oxidation or export. This is the case with an HFD. Histologically, when more than 50% of the liver cells have steatosis, this is termed NAFLD. In addition, hepatic steatosis is closely related to hepatic insulin resistance, endoplasmic reticulum, autistic autophagy, mitochondrial dysfunction, apoptosis, and inflammation (Chanclón et al., 2020; Loomba et al., 2021). Therefore, to observe the effect of MMQ-8 on the livers of obese rats, we performed tests on liver function, pathological changes, and inflammatory signaling pathways. As shown in Figures 4A–C, MMQ-8 did not influence liver weight, but it did decrease serum levels of AST and ALT compared with the model group. In contrast, each ST treatment group showed decreased liver weights while the serum levels of AST and ALT remained similar to the model group. H&E staining results (Figure 4E) showed that the model group had more hepatocyte steatosis, cytoplasmic vacuolation, ballooning, necrosis, and lobular inflammation than the control group. However, compared with the model group, the hepatocyte steatosis, cytoplasmic vacuolation and ballooning were significantly reduced, and there was lower necrotic or lobular inflammation in each MMQ-8 group. The pathological changes in each ST group were similar to the model group.

Meanwhile, we performed NAS on the liver H&E images and the scoring results are shown in Figure 4D. The NAS score of the model group was significantly higher than that of the control group. However, in each MMQ-8 group, NAS was significantly decreased compared with the model group. In ST groups, the NAS score was lower than in the model group, but there was no statistical difference. Moreover, to better observe hepatic steatosis, we performed liver oil red O staining and the results are shown in Figure 4F. Compared with the control group, the model group had significant fat deposition, while the MMQ-8 treatment group had significantly less fat deposition than the model group. The ST treatment group had the same results as the MMQ-8 group. Most importantly, we used the Western-blot method to detect the phosphorylation levels of the main regulatory proteins of the TNF signaling pathway, including IκBα, p38, ERK1/2, and SAPK/JNK in the liver. The results (Figures 4G–J) showed that the phosphorylation levels of IκBα, p38, ERK1/2, and SAPK/JNK proteins in the model group were significantly higher than for the control group. However, in the MMQ-8 and ST treatment groups, the phosphorylation levels of these proteins were significantly lower than those in the model group. These results demonstrated that MMQ-8 improved liver function and reduced hepatic steatosis and inflammation in obese rats. Moreover, MMQ-8 treatment showed no hepatotoxicity.

### MMQ-8 Decreased IL-6, TNF-α, and INOS, and Elevated IL-10 Level in Serum

**FIGURE 5:**
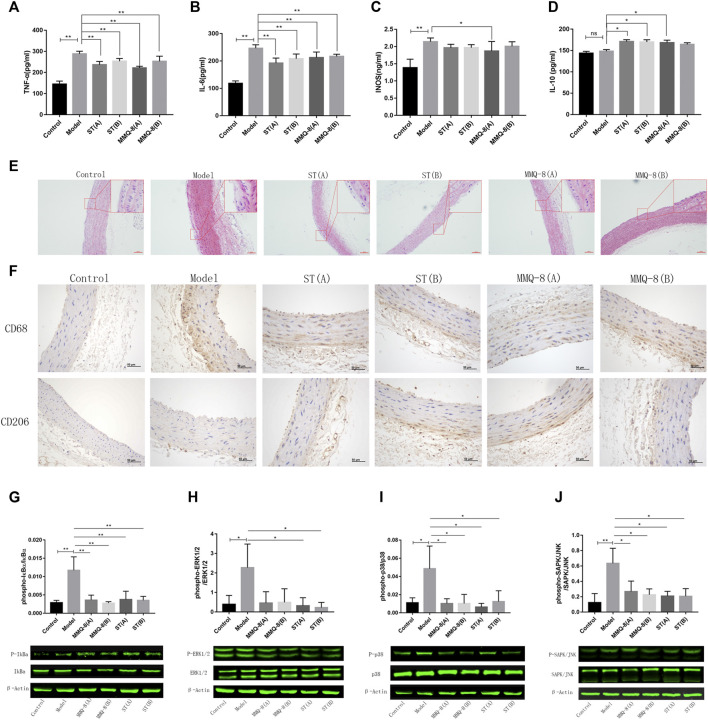
MMQ-8 treatment significantly inhibited inflammation and vascular injury. MMQ-8 treatment significantly decreased serum levels of pro-inflammatory factors TNF-α, IL-6, and INOS and elevated the serum level of anti-inflammatory factor IL-10. **(A)** Serum levels of TNF-α. **(B)** Serum levels of IL-6. **(C)** Serum levels of INOS. **(D)** Serum levels of IL-10. **(E)** MMQ-8 treatment reduced vascular endothelium damage, smooth muscle cell proliferation, and foam cell formation. Representative H&E staining of arteries (*n* = 6). **(F)** MMQ-8 decreased the number of macrophages and increased macrophage polarization to M2 in artery walls. Representative images of artery immunohistochemistry staining for CD68 and CD206 (*n* = 6). **(G)** MMQ-8 treatment decreased the phosphorylation levels of IκBα in blood vessels **(H)** MMQ-8 treatment decreased the phosphorylation levels of ERK1/2 in vasculature. **(I)** MMQ-8 treatment decreased the phosphorylation levels of p38 in vasculature. **(J)** MMQ-8 treatment decreased the phosphorylation levels of SAPK/JNK in vasculature. MMQ-8 reduced the vascular protein phosphorylation levels of the TNF signaling pathway in obese rats. Data are presented as means ± SD, *n* = 3, *p < 0.05, **p < 0.01.

Inflammation is also a key surveillance target for obesity. Compelling evidence reveals that white adipose tissue will secrete pro-inflammatory factors that induce a permanent state of systemic inflammatory response in obesity caused by an HFD (Xu et al., 2017). Therefore, we tested the serum levels of pro-inflammatory factors IL-6, TNF-α, and INOS, and anti-inflammatory factor IL-10. The results (Figures 5A–D) showed that these pro-inflammatory factors were significantly increased in the model group compared with the control group. In the MMQ-8 treatment group, the level of pro-inflammatory factors IL-6, TNF-α, and INOS was significantly decreased, and the level of anti-inflammatory factor IL-10 was increased compared with the model group. Compared with the model group, IL-6, and TNF-α were significantly reduced and the IL-10 was increased in each ST treatment group. These findings suggest that MMQ-8 has an anti-inflammatory action in obesity.

### MMQ-8 Inhibited Vascular Injury and Inflammation

Obesity is a main cause of AS. Although the mechanism of AS has not been fully revealed, it is generally believed that abnormal lipid metabolism, oxidative stress, and chronic inflammation caused by obesity lead to vascular endothelial damage and inflammation. Inflammatory cells such as mononuclear macrophages gather and infiltrate into the vascular wall, where they engulf large volumes of lipids, and then turn into foam cells. This vicious cycle and eventually forms multiple atherosclerotic plaques on the vascular wall (Kobiyama and Ley, 2018). Network analysis also suggests that MMQ-8 has certain therapeutic effects on AS. Therefore, to explore the effects of MMQ-8 on vascular injury and inflammation in greater detail, we first performed H&E staining on the aortic arches. As shown in Figure 5E, we observed vascular wall thickening, smooth muscle cell disturbance and migration, and foam cell formation in the model group compared with the control group. A small number of foam cells were present underneath endovascular cells in the MMQ-8 and ST groups compared with the model group.

Macrophages have an important role during the initiation and development of AS, and studies have shown that these differ with phenotype. CD68, a glycoprotein highly expressed by mononuclear phagocytes, is used as a marker for macrophages. Mannose receptor (CD206) is highly expressed in M2-like macrophages that play an anti-inflammatory role during inflammation (Nawaz et al., 2017). Thus, to observe the infiltration and phenotypic changes of macrophages in vascular walls, we next detected the expression of CD68 and CD206 by immunohistochemistry. As shown in Figure 5F, CD68 was significantly expressed in the model group, while CD206 was only slightly expressed compared with the control group. Furthermore, compared with the model group, the expression of CD68 in each ST and MMQ-8 group was reduced, while CD206 was significantly expressed in the ST(B) and MMQ-8(A) groups.

AS is a disease characterized by low-grade chronic inflammation of the arterial wall. Network analysis suggested that MMQ-8 significantly regulated inflammatory signaling pathways, including TNF, NF-κB, and MAPK. TNF is a classic, pleiotropic pro-inflammatory cytokine. It regulates cell survival, apoptosis, and differentiation, and promotes immune and inflammatory responses through the TNF receptor (TNFR1/2), activating multiple downstream signals transduction pathways, such as NF-κB, MAPK, and PI3K (Kalliolias and Ivashkiv, 2016; Ben et al., 2019). In addition, MMQ-8 significantly regulates the expression of serum inflammatory factors such as TNF-α, IL-6, INOS, IL-10, and the phenotype of macrophages. Therefore, to explore more details of the effect of MMQ-8 on vascular inflammation, we detected the phosphorylation levels of proteins in the TNF signaling pathway by Western-blot assay on aorta samples. These included IκBα, p38, ERK1/2, and SAPK/JNK. As shown in Figures 5G–J, compared with the control group, the phosphorylation levels of IκBα, p38, ERK1/2, and SAPK/JNK were significantly increased in the model group. In contrast, in the ST and MMQ-8 groups, the phosphorylation levels of these proteins were significantly lower than in the model group. These findings demonstrate that MMQ-8 inhibited vascular injury and inflammation.

### MMQ-8 Inhibited Bone Marrow Cell Inflammation

**FIGURE 6:**
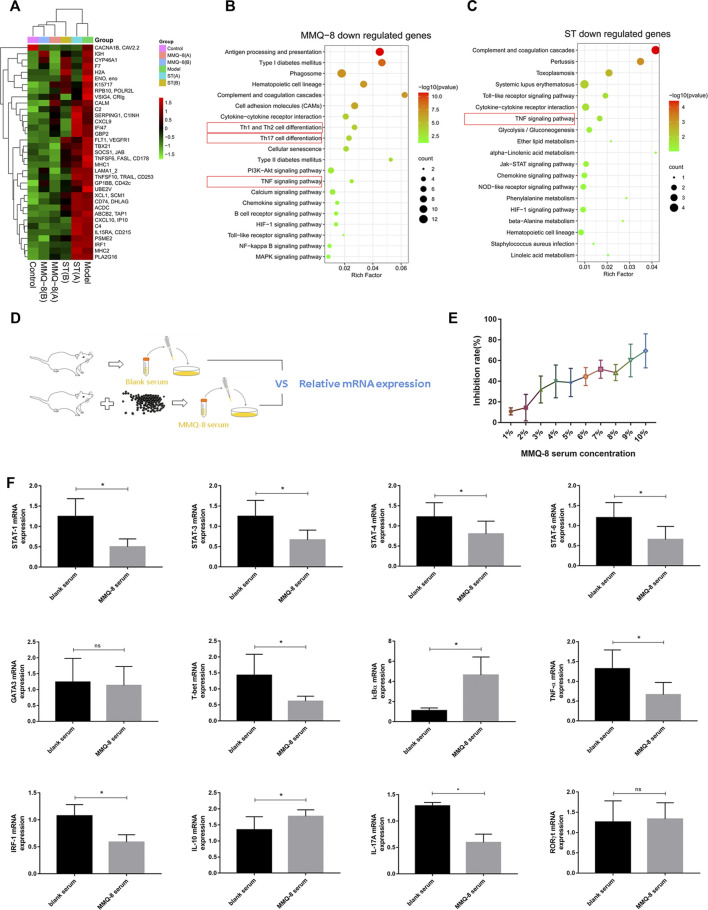
Continued

Bone marrow is the main site for blood cell formation. It is a primary lymphoid organ that supports lymphoid development and a host of naive and memory immune cells, plasma cells, regulatory T cells, and myeloid immune cells. The cellular niches of bone marrow can regulate hematopoietic stem cells (HSCs) and immune cell behavior through direct cell contact, growth factors and cytokines, and components of the extracellular matrix (Mercier et al., 2012; Liu et al., 2018). Studies have revealed that obesity can destroy bone marrow homeostasis and reduce the number of primitive HSCs (Hermetet et al., 2019). These findings suggest that bone marrow is closely related to the chronic inflammation induced by obesity. Therefore, to fully understand the regulatory effects of MMQ-8 on the bone marrow of obese rats, we performed bone marrow cell transcriptome sequencing. As shown in Figure 6A, using different expression analyses, we found that MMQ-8 treatment downregulated 35 genes that were different from the model group regarding immune response and lipid metabolism. Moreover, the expression of these 35 genes in the ST groups was no different than in the model groups, especially in the ST(A) group. We then performed KEGG enrichment analysis on these 35 genes and found that ST down-regulated genes that were different from the model group. As shown in Figure 6B, in the 35 MMQ-8 down-regulated genes, we obtained many inflammatory signaling pathways that were significantly enriched, including TNF signaling pathway, Th1 and Th2 cell differentiation, Th17 cell differentiation, and NF-κB. Although many inflammatory signaling pathways were down regulated in ST group, including TNF signaling pathway, toll-like receptor signaling pathway, and chemokine signaling pathway, the enrichment was not significant, as shown in Figure 6C. These results demonstrate that MMQ-8 inhibited inflammation in bone marrow cells in obese rats.

### MMQ-8 Inhibited the NF-κB Signaling Pathway and Th1, Th2, and Th17 Cell Differentiation Signaling Pathways *In Vitro*

Adaptive immune cells and innate immune cells play a key role in obesity-induced chronic low-grade inflammation. Many studies have shown that CD4^+^T cells exist in adipose tissue, NAFLD, and atherosclerotic plaques in obesity. These cells are implicated not only in inflammation but also in the development of insulin resistance and T2D. CD4^+^T cells mainly include Th1, Th2, Th17, and Treg cells. Of these, Th1 and Th17 cells are pro-inflammatory cells, while Th2 and Treg cells have the opposite effect. Th1 and Th17 cells promote macrophage migration and phenotypic differentiation to M1 by secreting inflammatory factors, while Th2 and Treg cells secrete anti-inflammatory factors such as IL-4, IL-10, and transforming growth factor-beta (TGF-β) (Kawasaki et al., 2012; McLaughlin et al., 2017). In our study, the network analysis and RNA sequencing of bone marrow cells in obese rats showed that MMQ-8 significantly regulated the signaling pathways involving NF-κB and Th1, Th2, and Th17 cell differentiation. Therefore, to further verify the effect of MMQ-8 on these signaling pathways, we used RT-qPCR to detect the effect of MMQ-8 drug-containing serum on the level of related mRNA in CTLL-2 cells *in vitro* (Figure 6D). The MMQ-8 serum concentration was 1% based on the inhibition rate (Figure 6E). Compared with normal rat serum, MMQ-8 medicated serum significantly lowered STAT1, STAT3, STAT4, STAT6, T- bet, TNF-α, IRF-1, and IL-17A mRNA levels; and significantly raised IκBα and IL-10 mRNA levels (Figure 6F). However, in each group, there was no significant difference in mRNA levels of RORγt and GATA3. T-bet, STAT1, and STAT4 are important in the differentiation process of CD4^+^T cells into Th1 cells. GATA3, STAT5, and STAT6 are the key to differentiation of CD4^+^T cells into Th2 cells, while RORγt and STAT3 are keys to the differentiation of CD4+T cells into Th17 cells (Kanhere et al., 2012). In addition, the activation of the NF-κB signaling pathway encourages the activation and differentiation of inflammatory T cells (Das et al., 2001). These results suggest that MMQ-8 has inhibitory effects on the NF-κB signaling pathway and the Th1, Th2, and Th17 cell differentiation signaling pathways. We demonstrated that MMQ-8 may potentially inhibit CD4^+^T cell differentiation into Th1 and Th17 cells, but more studies are needed to confirm this point.

## Discussion

Currently, statins are the primary choice for treating hyperlipidemia and preventing secondary diseases caused by obesity. However, there are still no radical treatments available. Therefore, greater attention is being paid to botanical drug studies. In a holistic and synergistic way, Chinese botanical drugs possess superior pharmacological properties for treating obesity (Chang et al., 2015; Chang et al., 2018). Our study revealed that MMQ-8 was able to optimize lipid metabolism and reduce inflammatory responses by inhibiting the TNF signaling pathway and the differentiation of Th1 and Th17 cells.

As a branch of TCM, Mongolian medicine has its own independent theoretical system and therapies. Mongolian medicine cures diseases by maintaining the balance of homeostasis *in vivo* in a holistic and synergistic way. The main sources of Mongolian medicines are botanical drugs (Qiburi et al., 2020). Botanical drugs are gaining increasing acceptance worldwide, for example, using Yin-xin-tong-mai, Huang-qi san, or Danning tablets. These TCMs have been shown to positively affect obesity (Hao et al., 2020; Ma et al., 2021; Zheng et al., 2021). The present study results also confirmed that MMQ-8 has positive effects on obesity.

The excessive fat deposition associated with obesity has multiple etiological factors but is widely considered the result of disequilibrium between energy intake and expenditure. As an energy source, the excessive intake of lipids is one of the main causes of obesity. Lipids absorbed into the body take the form of TG, cholesterol, and lipoids in the blood and are transported as VLDL, LDL, and HDL. VLDL and LDL are the main vehicles for carrying cholesterol to extra-hepatic tissues. At the same time, HDL plays the reverse role of cholesterol transport, carrying excess cholesterol from peripheral tissues to the liver (Pirillo et al., 2021). The liver is the main site of lipid metabolism, converting the cholesterol returned by HDL into bile acids for excretion. Furthermore, the liver is also one of the major sites for synthesizing triglycerides from the absorption of sugars; if these triglycerides are not transported away, NAFLD will develop (Friedman et al., 2018; Watt et al., 2019). Therefore, in addition to BMI, changes in blood lipid levels and liver pathology can also reflect lipid metabolism. In our study, MMQ-8 treatment not only reduced body weight, white adipose tissue weight, and liver steatosis, and also increased serum HDL levels. These results indicate that MMQ-8 can optimize lipid metabolism, but the mechanism by which this takes place still needs further research.

Nowadays, it is widely considered that obesity is a type of inflammatory disease. Studies indicate that chronic low-grade inflammation induced by obesity is systemic, including multiple organs such as adipose tissue, the pancreas, liver, skeletal muscle, artery, heart, and brain. This chronic inflammation is closely associated with insulin resistance, AS, T2D, and the development of some cancers (Chen et al., 2018). It involves the activation of multiple inflammatory signaling pathways, such as the T cell receptor signaling pathway, NF-κB, PI3K, and TNF and the secretion of many pro-inflammatory cytokines, e.g., TNF-α, IL-6, INOS, and monocyte chemoattractant protein 1 (McP-1), and IL-1 (McLaughlin et al., 2017; Malik et al., 2020; Wu and Ballantyne, 2020). In our study, network analysis showed that MMQ-8 significantly regulated these inflammatory signaling pathways. At the same time, *in vivo* experiments, demonstrated that MMQ-8 can significantly reduce the activation of TNF signaling pathways in the liver and blood vessels. It also decreased secretion of the pro-inflammatory factors TNF-α, IL-6, and INOS. In addition, MMQ-8 increased the levels of anti-inflammatory factor IL-10 in serum. These results show that MMQ-8 has wide-ranging anti-inflammatory effects.

Inflammation induced by obesity is one of the main causes of the occurrence and development of AS. In obesity, the increasing levels of LDL, triglycerides, and inflammatory factors in circulating blood cause hemodynamic changes and an oxidative stress reaction. These induce dysfunction and an inflammatory reaction in the vascular endothelium. Then lipids and inflammatory cells infiltrate vascular walls. Macrophages are the main inflammatory cells involved in this process. After infiltrating vascular walls, the macrophages engulf lipids and turn into foam cells, causing inflammation of vessels. This creates a vicious cycle of inflammation and foam cell accumulation, eventually leading to AS (Kobiyama and Ley, 2018; Xu et al., 2019; Barrett, 2020). During the pathological process of AS, the polarization of macrophages to M1 will promote the local inflammatory response of blood vessels, while polarization to M2 will have the opposite effect. M2-type macrophages can promote the repair of damaged blood vessels and the stabilization of plaques. Studies have shown that CD68^+^CD206^+^M2 phenotype macrophages are a main sub-type of macrophage, which play an anti-inflammatory role in inflammatory diseases (Nawaz et al., 2017). In our study, MMQ-8 inhibited endothelial injury and macrophage infiltration, and promoted the polarization of macrophages to M2. In addition, MMQ-8 significantly decreased the protein phosphorylation of IκBα, p38, ERK1/2, and SAPK/JNK in the TNF signaling pathway in the aorta. TNF, as a classical pleiotropic pro-inflammatory cytokine, is produced by a range of immune and non-immune cells and is the first cytokine to appear within minutes of any injury or stress caused by pro-inflammatory stimuli. TNF activates the downstream signaling pathway, including NF-κB, MAPK, and PI3K through binding with TNFR1 and TNFR2 to regulate cell survival and inflammation. Therefore, this signaling pathway is closely related to metabolic inflammation, AS, and insulin resistance (Kalliolias and Ivashkiv, 2016; Dou et al., 2018; Jaiswal et al., 2019). It can therefore be understood that MMQ-8 inhibited vascular injury and macrophage polarization. Moreover, MMQ-8 played a vital role in metabolic inflammation *via* down-regulating the TNF signaling pathway.

Both adaptive and innate immunity play important roles in obesity-induced systemic inflammation (McLaughlin et al., 2017; van der Zalm et al., 2020). HSCs are a source of leukocytes critical to innate and adaptive immunity. It has been observed that traces of immune cells are activated in bone marrow, including CD4^+^T cells, CD8^+^T cells, CD11c+ dendritic cells (DCs), B cells, plasma cells, NKT cells, mesenchymal stem cells (MSCs), and myeloid-derived suppressor cells (MDSCs) (Mercier et al., 2012; Hermetet et al., 2019). Mercier et al. (2012) and Hermetet et al. (2019) detected a loss of half of the most primitive HSC in bone marrow cells of HFD-fed mice. Moreover, Liu et al. (2018) found that obese mice exhibit poor emergency immune responses in a toll-like receptor 4-dependent manner. These studies indicate that the bone marrow, an immune regulatory organ, is closely related to the inflammation induced by obesity. In our study, RNA sequencing results of bone marrow cells showed that MMQ-8 inhibited TNF, the NF-κB signaling pathway, and Th1, Th2, and Th17 cell differentiation signaling pathways. In addition, it was further confirmed *via in vitro* experiments. These findings revealed that the TNF signaling pathway and CD4+T cell differentiation were necessary to promote the effect of MMQ-8. In network analysis, we observed 37 active metabolites of MMQ-8 directly regulating these signaling pathways.

In summary, we determined the pharmacological actions and relevant metabolites of MMQ-8 in obesity for the first time. Our study revealed that MMQ-8 can optimize lipid metabolism and reduce chronic inflammation in obesity. Its anti-inflammatory effect was accomplished by promoting macrophage polarization to M1, reducing macrophage infiltration, and inhibiting the signaling pathways regarding TNF, NF-κB, Th1, and Th2 cell and Th17 cell differentiation. However, we focused only on pharmacodynamic evaluation and relevant metabolites of MMQ-8. There remains a great deal of in-depth research to be done, for example, to understand the principle of compound compatibility and the inhibition effects on hepatic steatosis, T cell differentiation, and inflammatory signal transduction. We believe that it is necessary to continue exploring these potential mechanisms of MMQ-8.

## Data Availability

The datasets presented in this study can be found in online repositories. The names of the repository/repositories and accession number(s) can be found below: https://www.ncbi.nlm.nih.gov/bioproject/, PRJNA820308.

## Glossary

ALT: Alanine aminotransferase
AS: Atherosclerosis
AST: Aspartate aminotransferase
CCL2: C-C motif chemokine 2
CTLL-2: IL-2-dependent cell line
CVD: Cardiovascular diseases
ELISA: Enzyme-linked immunosorbent assay
ERK1/2: Extracellular regulated kinase 1/2
GATA3: GATA binding protein 3
H&E: Hematoxylin and eosin
HDL: High-density lipoprotein
HFD: High fat diet
HIF-1: Hypoxia-inducible factor 1
IkBα: nuclear factor-kappa B inhibitor alpha
IKKβ: I kappa B kinase beta
IL: Interleukin
INOS: Inducible Nitric Oxide Synthase
IRF-1: Interferon regulatory factor 1
JNK: c-Jun N-terminal kinase
LDL: Low-density lipoprotein
MMQ-8: Traditional Mongolian Medicine Qiqirigan-8
MAPK: mitogen-activated protein kinase
NAFLD: Non-alcoholic fatty liver disease
NAS: NAFLD activity scores
NEG: Negative
NF-kB: Nuclear factor-kappa B
p38: Mitogen-activated protein kinase
PI3K: Phosphatidylinositol 3-kinase
PPAR: peroxisome proliferator-activated receptor
POS: Positive
RORγt: Retinoid-related orphan receptor-gamma-t
RT-qPCR: Quantitative real-time polymerase chain reaction
SAPK: Stress-activated protein kinase
ST: Simvastatin
STAT: Signal transducer and activator of transcription
T2D: Type 2 diabetes
TC: Total cholesterol
TCM: Traditional Chinese Medicine
TG: Triglyceride
TGF-β: Transforming growth factor-beta
Th: T-helper
TNF: Tumor necrosis factor
TNFR1/2: TNF receptor 1/2
UHPLC-QE-MS: Ultrahigh-performance liquid chromatograph Q extractive mass spectrometry
